# A prediction nomogram for faltering catch-up growth in full-term small-for-gestational-age infants: a retrospective cohort study

**DOI:** 10.3389/fped.2026.1792285

**Published:** 2026-05-18

**Authors:** Qian Hu, Sufei Yang, Ping Li, Jiawei Luo, Fan Yang

**Affiliations:** 1 Department of Pediatrics, West China Second University Hospital, Sichuan University, Chengdu, China; 2 Department of Ministry of Education, Key Laboratory of Birth Defects and Related Diseases of Women and Children, Sichuan University, Chengdu, China; 3 West China Biomedical Big Data Center, West China Hospital/West China School of Medicine, Sichuan University, Chengdu, China

**Keywords:** birth length *Z* score, birth weight *Z* score, catch-up growth, prediction nomogram, small-for-gestational-age, target height *Z* score

## Abstract

**Background and objective:**

Catch-up growth (CUG) before two years of age in small-for-gestational-age (SGA) infants is a key predictor of adult short stature. We aimed to establish and temporally validate a prediction nomogram for faltering CUG (FCUG) in full-term SGA infants before two years of age.

**Methods:**

We conducted a retrospective cohort study of full-term SGA infants at West China Second University Hospital, with cohorts defined as: development (January 1996–December 2019) and temporal validation (January 2020–July 2023). Full-term SGA infants, defined by birth weight or length below 10th percentile per INTERGROWTH-21st standards (*n* = 1,185 in development; *n* = 294 in validation). FCUG was defined as failure to increase length-for-age *Z* score by ≥0.67 above birth *Z* score by 24 months. Sex, birth weight Z, birth length Z and target height *Z* scores were entered into a multivariable logistic model. Discrimination (AUC, sensitivity, specificity), calibration (Hosmer–Lemeshow, calibration plot), internal (1,000 bootstrap resamples) and temporal (2020–2023 cohort) validations were performed.

**Results:**

In the development cohort, 280 of 1,185 (23.6%) of SGA infants experienced FCUG before age two, compared with 60 of 294 (20.4%) in the temporal validation cohort. In multivariable logistic regression analyses, male sex, lower birth length *Z* score, lower birth weight *Z* score, and lower target height *Z* score were all significantly associated with FCUG (all *p* < 0.05). These four predictors were incorporated into a nomogram. In the development cohort, the model demonstrated excellent discrimination (AUC = 0.810, 95% CI: 0.785–0.835) with a sensitivity of 74% and specificity of 74% at the optimal cut-off. Bootstrap validation (1,000 resamples) confirmed a stable AUC of 0.810. When applied to the temporal cohort, the nomogram achieved an AUC of 0.784 (95% CI: 0.730–0.838), with sensitivity of 93% and specificity of 56%.

**Conclusions:**

We have developed and temporally validated a clinically applicable nomogram that reliably identifies full-term SGA infants at high risk of failing to achieve catch-up growth by age two. With robust discrimination and calibration, this tool can support early risk stratification and guide targeted nutritional or developmental interventions.

## Introduction

Small-for-gestational-age (SGA) is typically defined as birth weight and/or birth length below the 10th percentile for gestational age (1). A multicenter study from developed countries reported an SGA incidence of 11.3% (2). The overall incidence of SGA in 30 provinces of China was 6.4% from 2012 to 2020 (3), while it was 12.28% in Guangdong Province, China, from 2014 to 2019 (4). Although most SGA infants achieve spontaneous catch-up growth (CUG) by age two, approximately 10% experience faltering catch-up growth (FCUG) and remain at risk for adult short stature (5, 6). CUG in early childhood is crucial for SGA infants to achieve normal adult height (6). SGA infants who fail to attain catch-up growth by two years old are less likely to do so later (7). Despite its clinical significance, few studies have systematically examined risk factors for FCUG in SGA infants (8). Nomograms—graphical calculators that integrate multiple predictors—have become valuable tools for individualized risk estimation in various clinical contexts (9, 10).

Therefore, this study was designed with dual objectives: (1) to identify perinatal and familial risk factors predicting FCUG in SGA infants within the first two years of life, and (2) to develop and temporally validate a practical nomogram for early identification of infants at high risk of FCUG. By enabling targeted monitoring and intervention within the critical growth window, this tool seeks to improve long-term stature outcomes for SGA infants.

## Materials and methods

### Study design

This was a retrospective cohort study based on the SGA infant cohort established at the West China Second University Hospital of Sichuan University. The study protocol was approved by the medical ethics committee of the West China Second University Hospital of Sichuan University with approval number 2019YFC0840702. Written informed consent was obtained from all parents.

### Study population

The prediction model was developed using a development cohort comprising SGA infants systematically enrolled at West China Second University Hospital of Sichuan University between January 1996 and December 2019. For temporal validation, we established a cohort of prospectively followed SGA infants who underwent standardized growth surveillance at our institution from January 2020 through July 2023, ensuring chronological separation from model development data.

Infants were included in the study if they met all of the following criteria: 1) had a birth weight and/or birth length below the 10th percentile based on the INTERGROWTH-21st project (11); 2) had a gestational age ≥37 weeks; 3) were born from singleton pregnancies; and 4) known malformations, inherited metabolic diseases and chromosomal diseases were excluded. We excluded infants with missing birth length data or those who did not complete CUG but were lost to follow-up before two years of age.

According to whether they experienced CUG before two years of age, SGA infants were divided into successful catch-up growth (SCUG) group and FCUG group. Risk factors for FCUG in the development cohort were analyzed using logistic regression with FCUG as the dependent variable, and a predictive nomogram was subsequently constructed.

### Data collection

We developed a standardized database to prospectively capture multidimensional clinical variables for SGA infants, encompassing demographics, underlying diseases and physical indicators, and their parental physical indicators. The physical indicators of the SGA infants were measured and recorded by two trained nurses at a regular rate: once a month until six months after birth, once every two months between six months and one year of age, and once every three months between one and two years of age. Weight was measured by an electronic scale (Seca376; Seca Measuring System Co., Ltd., Germany). Length was measured in the supine position by a measurement bed with a footboard (Seca416; Seca Measuring System Co., Ltd., Germany). Weight and length were separately assessed to the nearest 100 g and 0.1 cm. The measuring instruments were calibrated regularly.

### Definitions

Asymmetric SGA was defined as a weight/length (W/L) ratio at birth below the 10th percentile. The reference values for W/L were derived from the INTERGROWTH-21st project (12). Whether the birth weight or birth length was below the 3rd percentile based on the INTERGROWTH-21st project (11). Birth weight *Z* score (BWZ), birth length *Z* score (BLZ), weight-for-age *Z* score (WAZ), length-for-age *Z* score (LAZ), and target height *Z* score (THZ) values were calculated according to the 2006 World Health Organization (WHO) Child Growth Standards. *Z* scores between −6 and 6 were considered valid, and values outside this range were discarded (13, 14). WAZ and LAZ values were used to establish growth trajectories for female and male SGA infants in development cohort, respectively. The target height (TH) was calculated using the mid-parental height method: Boys: TH = [(father's height + mother's height) + 13 cm] ÷ 2; Girls: TH = [(father's height + mother's height) − 13 cm] ÷ 2 (15). FCUG was defined as the absence of any point before two years of age at which the change in LAZ relative to BLZ exceeded 0.67 (5).

### Statistical analysis

Analyses were performed using R v4.1.1. Continuous variables—non-normally distributed—are reported as medians [interquartile range (IQR)]; categorical variables as counts (%). Comparisons between the development cohort and temporal validation cohort were performed using the Mann–Whitney *U* test for continuous data and *χ*^2^ test for categorical data.

Missing covariate data were handled by multiple imputation (16). Univariate logistic regression screened candidate predictors, which—together with clinically relevant variables—were entered into a forward–backward stepwise multivariable logistic model minimizing the Akaike information criterion (AIC). Model comparisons employed likelihood ratio tests, and variance inflation factors (VIFs) assessed multicollinearity (VIF > 10) (10). Clinically plausible interactions were examined.

Model performance was evaluated in both the development and temporal validation cohorts. Discrimination was assessed by the area under the ROC curve (AUC), with sensitivity and specificity determined at the Youden index. Calibration was examined via calibration plots and the Hosmer–Lemeshow test. Internal stability was appraised by 1,000 bootstrap resamples, and clinical utility by decision curve analysis. A nomogram was constructed to visualize the final model. Statistical significance was set at two-sided *P* < 0.05.

## Results

### Baseline characteristics and growth trajectories

**Figure 1:**
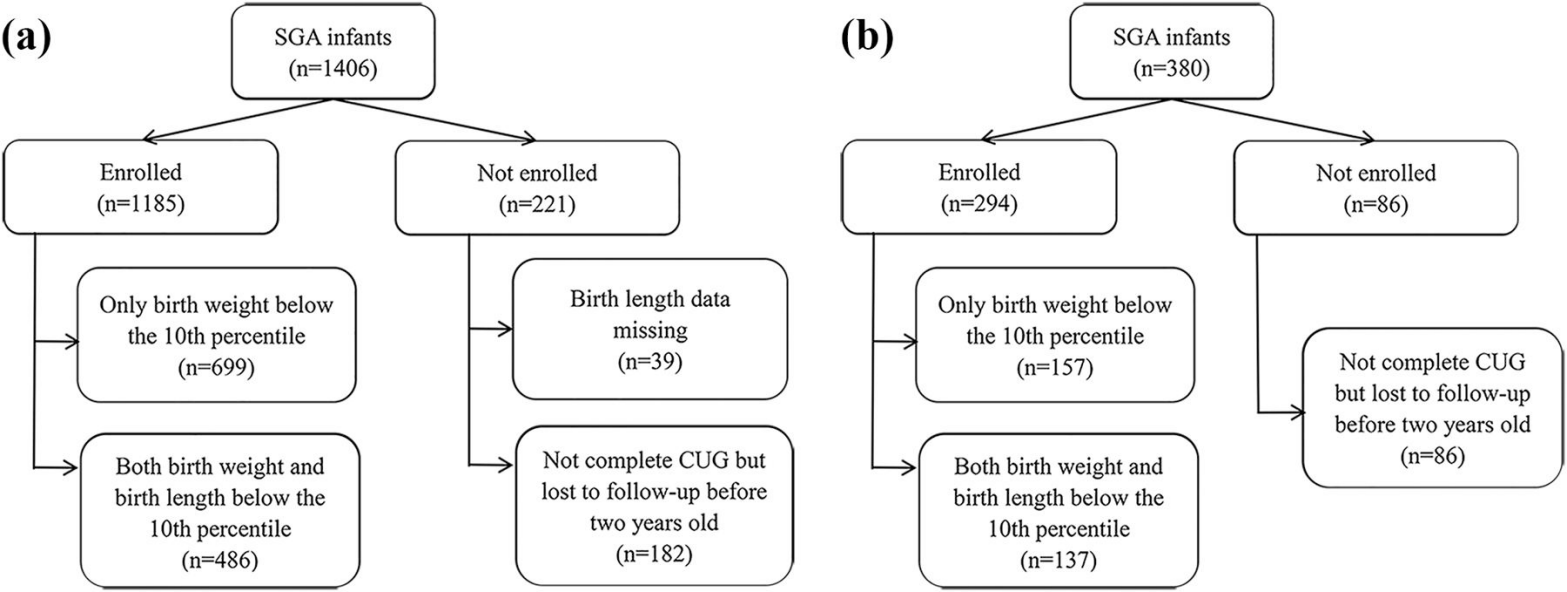
**(a)** Flowchart of SGA infants enrolled in and excluded from the study in development cohort; **(b)** flowchart of SGA infants enrolled in and excluded from the study in temporal validation cohort. SGA, small-for-gestational-age; CUG, catch-up growth.

In development cohort, a total of 1,406 term singleton SGA infants were initially enrolled; 39 lacked birth length data and 182 were lost to follow-up before two years, leaving 1,185 for analysis (Supplementary Table S1). Of these, 486 had both birth weight and birth length below the 10th percentile, while 699 had birth weight below the 10th percentile but length at or above the 10th percentile (Figure 1a). In the temporal validation cohort, 380 term singleton SGA infants were enrolled; 86 were lost before two years, leaving 294 infants for analysis (Figure 1b; Supplementary Table S1).

**Figure 2:**
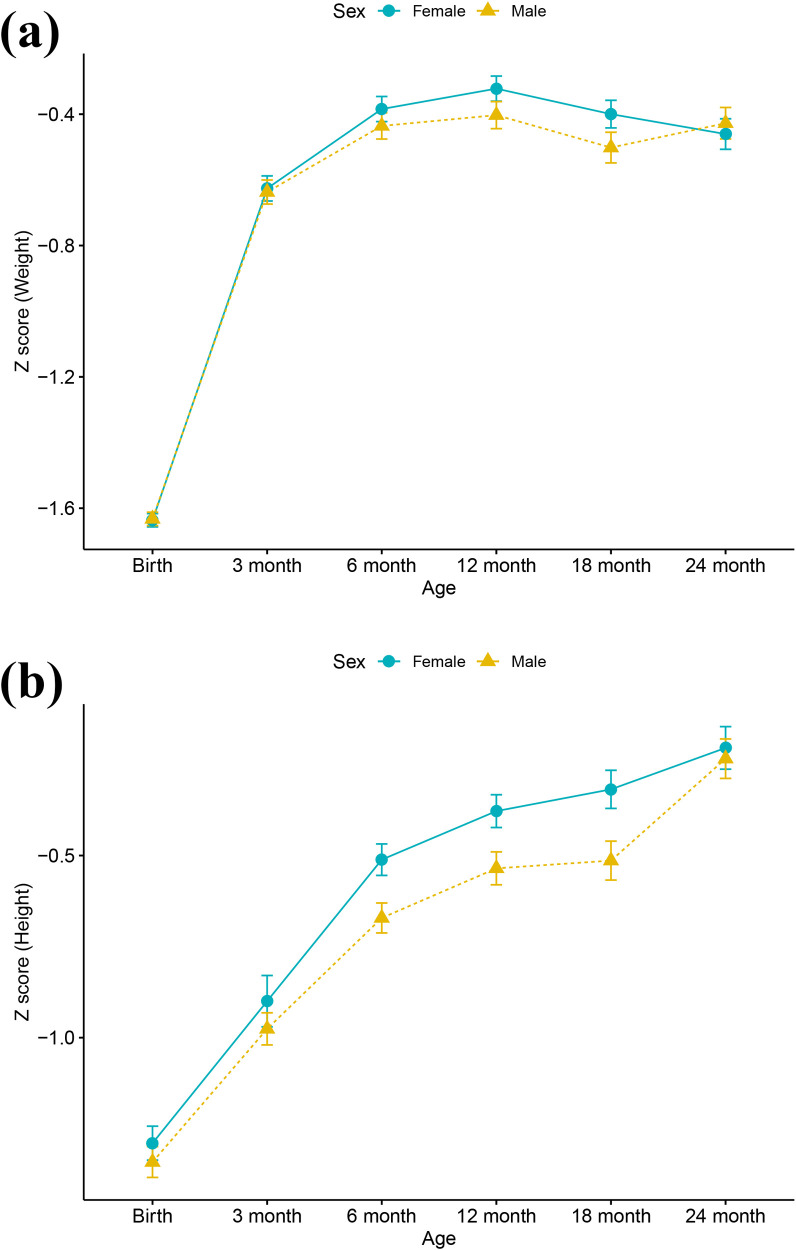
**(a)** Weight-for-age *Z* score trajectories for SGA infants from birth to 24 months; **(b)** length-for-age *Z* score trajectories for SGA infants from birth to 24 months. SGA, small-for-gestational-age.

In the development cohort, WAZ increased sharply from a median of −1.62 at birth to −0.70 by 3 months, peaking around 12 months, with the greatest gains occurring within the first six months (Figure 2a). LAZ rose more gradually from −0.85 at birth to −0.50 by 6 months and continued to improve through 24 months (Figure 2b). Across both measures, female infants consistently exhibited faster catch-up in length than male infants. Ultimately, a total of 280/1,185 (23.61%) and 60/294 (20.4%) SGA infants experienced FCUG by two years of age, respectively in development cohort and in temporal validation cohort (Supplementary Table S2).

### Comparison of development and temporal validation cohorts

Compared with the development cohort, infants in the temporal validation cohort had significantly lower BWZ and BLZ values, but higher parental HAZ, THZ values and THZ–BLZ differentials (Table 1). In contrast, critical neonatal characteristics including sex distribution, symmetry status, and growth restriction severity showed no statistically significant disparities between cohorts (all *P* > 0.05).

**Table 1:** Characteristics of SGA infants in the development and temporal validation cohort.

Variables	Level	Development cohort	Temporal validation cohort	*P*
*n*		1,185	294	
Gestational age		273.00 [267.00, 280.00]	273.00 [266.25, 280.00]	0.375
BWZ		−1.55 [−1.95, −1.27]	−1.71 [−2.04, −1.36]	0.001**
BLZ		−1.15 [−2.05, −0.62]	−1.52 [−2.18, −1.00]	0.007**
Mother's HAZ		−0.49 [−1.10, −0.18]	−0.49 [−0.87, −0.03]	0.034*
Father's HAZ		−0.75 [−1.16, −0.34]	−0.62 [−0.89, −0.21]	0.01*
THZ		−0.68 [−1.03, −0.34]	−0.49 [−0.89, −0.21]	0.001**
THZ—BLZ		0.59 [−0.10, 1.43]	0.97 [0.20, 1.64]	<0.001***
Male sex (%)	No	605 (51.1)	147 (50.0)	0.796
	Yes	580 (48.9)	147 (50.0)	
Symmetric (%)	No	1,034 (87.3)	248 (84.4)	0.224
	Yes	151 (12.7)	46 (15.6)	
BL < P10 (%)	No	699 (59.0)	157 (53.4)	0.095
	Yes	486 (41.0)	137 (46.6)	
BL < P3 (%)	No	938 (79.2)	231 (78.6)	0.888
	Yes	247 (20.8)	63 (21.4)	
BW < P3 (%)	No	915 (77.2)	227 (77.2)	1
	Yes	270 (22.8)	67 (22.8)	
FCUG (%)	No	905 (76.4)	234 (79.6)	0.273
	Yes	280 (23.6)	60 (20.4)	

SGA, small-for-gestational-age; FCUG, faltering catch-up growth; BWZ, birth weight *Z* score; BLZ, birth length *Z* score; HAZ, height-for-age *Z* score; THZ, target height *Z* score; THZ—BLZ, the difference between the target height *Z* score and birth length *Z* score; BW, birth weight; BL, birth length.

* Significant differences with *P* < 0.05.

** Significant differences with *P* < 0.01.

*** Significant differences with *P* < 0.001.

### Model development

FCUG was operationalized as the primary endpoint. Using the development cohort, we initiated model development through univariate logistic regression analysis. Initial screening revealed that all candidate predictors except male sex demonstrated statistically significant associations with FCUG. However, given the well-established clinical relevance of sex in growth patterns, sex was retained in the multivariable logistic regression model. The result revealed that male sex, BLZ, BWZ and THZ values were predictors for FCUG in SGA infants (Table 2). Model 1 was built based on the above predictors. Notably, the coefficient for BWZ in Model 1 was positive, contrary to its negative association in the univariate analysis. Meanwhile, based on clinical observations suggesting potential interaction effects between BLZ and BWZ, we incorporated a BLZ:BWZ interaction term into Model 2 after mean-centering these variables. Model 2 demonstrated improved performance, evidenced by a lower AIC value compared to Model 1 and a statistically significant likelihood ratio test, confirming superior model fit (Supplementary Table S3). The VIF values of male sex, BLZ, BWZ, THZ, and BLZ:BWZ were 1.02, 2.13, 1.70, 1.12 and 1.24, respectively, indicating that there was no severe multicollinearity. By setting FCUG as 1 and SCUG as 0, the multivariate logistic regression model was as follows: Log (odds of FCUG) = 0.40824 × Male sex + 1.57924 × BLZ − 0.87197 × BWZ − 1.01733 × THZ + 0.46651 × BLZ:BWZ − 2.66329, where FCUG indicated faltering catch-up growth; BLZ indicated Birth length *Z* scores; BWZ indicated Birth weight *Z*; THZ indicated Target height *Z* scores.

**Table 2:** Logistic regression analysis to establish a prediction model for FCUG in SGA infants.

Variables	Univariate analysis			Multivariate analysis		
	Estimate	OR	*P*	Estimate	OR	*P*
Gestational age	0.055	1.06 (1.04–1.08)	<0.001***			
BWZ	0.864	2.37 (1.77–3.23)	<0.001***	−0.87197	0.418 (0.276–0.626)	<0.001***
BLZ	1.146	3.15 (2.62–3.82)	<0.001***	1.57924	4.851 (3.821–6.243)	<0.001***
Mother's HAZ	−0.285	0.75 (0.62–0.91)	0.003**			
Father's HAZ	−0.397	0.67 (0.55–0.82)	<0.001***			
THZ	−0.576	0.56 (0.44–0.72)	<0.001***	−1.01733	0.362 (0.269–0.482)	<0.001***
THZ—BLZ	−1.207	0.30 (0.25–0.36)	<0.001***			
Male sex	0.168	1.18 (0.90–1.55)	0.221	0.40824	1.504 (1.104–2.055)	0.00993**
Symmetric	−1.499	0.22 (0.11–0.40)	<0.001***			
BL < P10	−1.633	0.20 (0.14–0.27)	<0.001***			
BL < P3	−1.969	0.14 (0.07–0.24)	<0.001***			
BW < P3	−0.39	0.68 (0.48–0.95)	0.025*			

SGA, small-for-gestational-age; FCUG, faltering catch-up growth; BWZ, birth weight *Z* score; BLZ, birth length *Z* score; HAZ, height-for-age *Z* score; THZ, target height *Z* score; THZ—BLZ, the difference between the target height *Z* score and birth length *Z* score; BW, birth weight; BL, birth length.

* Significant differences with *P* < 0.05.

** Significant differences with *P* < 0.01.

*** Significant differences with *P* < 0.001.

### Model evaluation

**Figure 3:**
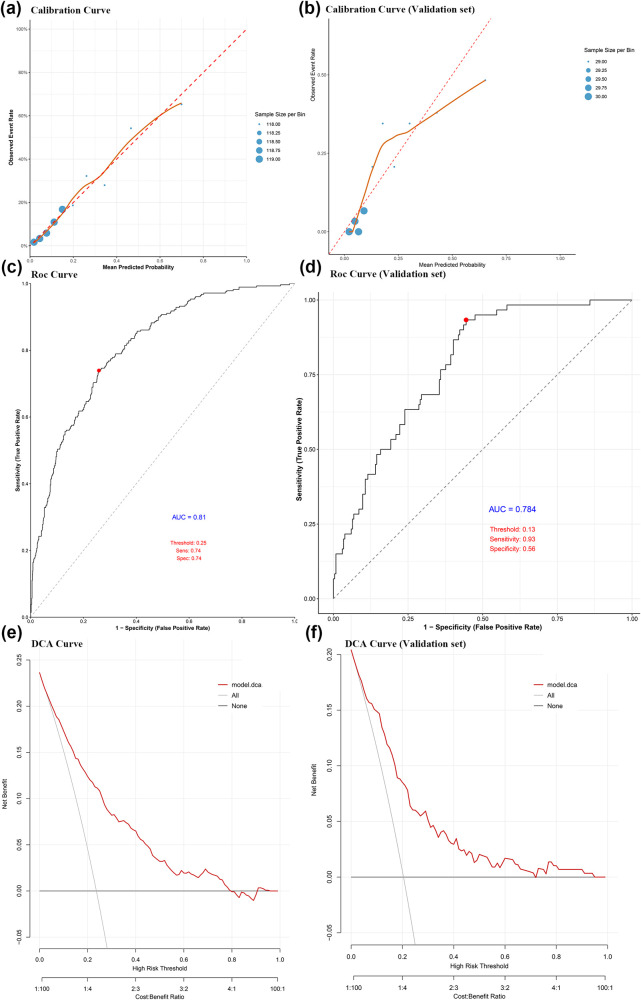
**(a)** The calibration curve of the model predicting the occurrence of FCUG in development cohort; **(b)** the calibration curve of the model predicting the occurrence of FCUG in temporal validation cohort; **(c)** the ROC curve of the model predicting the occurrence of FCUG in development cohort; **(d)** the ROC curve of the model predicting the occurrence of FCUG in temporal validation cohort; **(e)** the decision curve of the model predicting the occurrence of FCUG in development cohort; **(f)** the decision curve of the model predicting the occurrence of FCUG in temporal validation cohort. FCUG, faltering catch-up growth; AUC (the area under the ROC curve); DCA (decision curve analysis).

**Figure 4:**
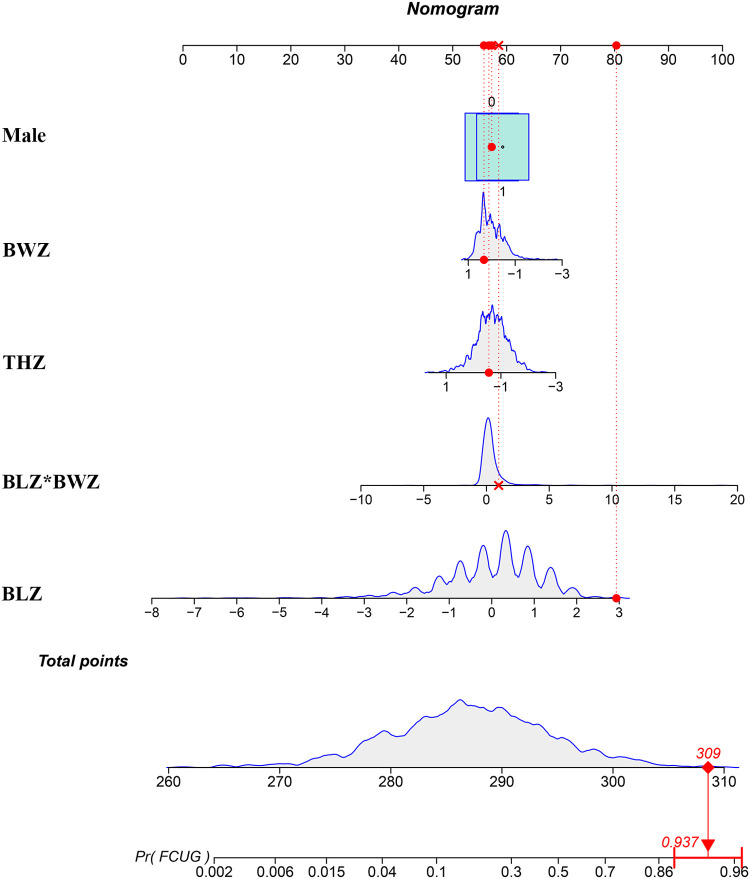
Nomogram of the model predicting the occurrence of FCUG in SGA infants. FCUG, faltering catch-up growth; SGA, small-for-gestational-age; THZ, target height *Z* score; BLZ, birth length *Z* score; BWZ, birth weight *Z* score.

Calibration analysis demonstrated satisfactory calibration fidelity across cohorts. In the development cohort, the Hosmer–Lemeshow test yielded a non-significant *P*-value of 0.299, while the temporal validation cohort showed borderline significance (*P* = 0.055), indicating acceptable model calibration. As visualized in Figures 3a,b, the calibration curves revealed optimal agreement between predicted and observed probabilities in the low-risk stratum (<20% FCUG probability), with systematic overestimation or underestimation emerging in higher-risk scenarios (>20%). These findings collectively suggest that the nomogram provides robust predictive accuracy for SGA infants with baseline FCUG risk below 20%, while warranting cautious interpretation for high-risk subgroups. The ROC analysis in the development cohort demonstrated robust discriminative capacity, with an AUC of 0.810, sensitivity of 0.74, and specificity of 0.74 at the Youden's index-optimized threshold (Figure 3c). Temporal validation yielded an AUC of 0.784, sensitivity of 0.93, and specificity of 0.56 (Figure 3e), corroborating generalizability. Internal validation via bootstrap resamples maintained an AUC of 0.810, indicating stable performance. DCA further demonstrated the clinical utility of the model. Across a range of clinically relevant risk thresholds, the model provided a greater net benefit compared to both “treat-all” and “treat-none” strategies (Figures 3e–f). To facilitate bedside application, we developed a nomogram for risk stratification (Figure 4).

## Discussion

In this retrospective cohort study, we developed and validated a prediction nomogram for faltering catch-up growth in term SGA infants within the first two years of life. Approximately one-quarter of infants in the development cohort and one-fifth in the temporal validation cohort met our predefined criterion for FCUG. Male sex, lower birth length *Z* score, lower birth weight *Z* score, and lower target height *Z* score emerged as independent predictors, highlighting the combined influence of perinatal anthropometry and genetic growth potential. The nomogram demonstrated robust discrimination and calibration, with an AUC exceeding 0.80 in the development cohort and acceptable performance in the external temporal validation cohort. These findings suggest that early postnatal risk stratification for FCUG is feasible and may provide clinicians with a practical tool to identify high-risk infants who warrant closer monitoring and potentially early interventions.

The incidence of FCUG in our study (23.6% in the development cohort and 20.4% in the temporal validation cohort) lies at the higher end of the range reported in previous literature (5).

Earlier studies that defined catch-up growth using threshold-based criteria, such as achieving weight- or length-for-age above −2 SD or the 3rd percentile by 24 months, generally reported lower rates of FCUG, around 10%–20% (17, 18). In contrast, studies employing velocity-based definitions, which require a meaningful upward shift in growth trajectory, often documented substantially higher proportions (19). Our definition—failure to exceed a 0.67 increase in LAZ relative to BLZ before 24 months—falls into this velocity-based category, which may partly explain the higher incidence observed. These discrepancies underscore the substantial impact of heterogeneous definitions of CUG on reported outcomes and highlight the urgent need for standardized criteria to facilitate cross-study comparisons and clinical translation.

An important consideration is which definition of catch-up growth is most appropriate for predicting long-term outcomes in SGA infants. Threshold-based definitions, such as reaching a weight- or length-for-age above −2 SD or the 3rd percentile, are simple, clinically intuitive, and consistent with the diagnostic criteria for stunting (17, 18). However, they may overlook infants whose growth velocity remains suboptimal despite achieving an apparently adequate anthropometric level. In contrast, velocity-based definitions, such as the threshold of a 0.67 *z*-score increment adopted in this study, exhibit higher sensitivity to early growth dynamics. This approach enhances the identification of infants at risk of persistent growth impairment or adverse metabolic outcomes. Concurrently, this accelerated growth pattern suggests that the original constraints limiting growth, such as potentially nutritional, placental, or metabolic, have been alleviated, thereby enabling a period of catch-up growth in SGA infants (20). Nevertheless, velocity-based criteria may classify a larger proportion of infants as having FCUG, which complicates comparisons across studies and may reduce clinical feasibility. Taken together, while both approaches have value, velocity-based definitions may be more informative for early risk stratification and prognosis. In contrast, threshold-based definitions may be more practical for routine clinical and public health monitoring. Future work linking these definitions to long-term outcomes will be essential to determine the most clinically meaningful standard.

The temporal validation cohort demonstrated significantly lower BWZ and BLZ compared to the development cohort. This likely reflects the advancements in perinatal medicine and neonatal intensive care medical technology in China in recent years (21). Furthermore, the validation cohort exhibited greater parental HAZ and THZ exceeding those in the development cohort. These findings align with global secular growth trends documented by the NCD Risk Factor Collaboration (22), which reported that between 1985 and 2019, Chinese males exhibited the world's most rapid height velocity, while females ranked third globally. This generational acceleration is attributed to synergistic improvements in early-life nutrition and socioeconomic development. Nevertheless, based on the model evaluation results, these observed discrepancies demonstrated minimal impact on predictive accuracy.

By analyzing the risk factors for FCUG in full-term SGA infants, we found that male infants had an almost 1.5-fold higher risk of FCUG than female infants, which was similar to the findings of Sinha et al. (23). This may be related to the male disadvantage in linear growth during early childhood (14). Consistent with the findings of Luo et al., we found that the higher the BLZ values or the lower the THZ values, the more SGA infants were prone to FCUG (8). At the same time, Karlberg et al. also found that BLZ and THZ values were significantly associated with an increase in HAZ values from birth to 18 years of age in SGA infants (6). BWZ was identified as an independent protective factor against FCUG in SGA infants. However, this protective association was modified by BLZ. This may partly explain why van Wyk et al. reported a paradoxically greater propensity for catch-up growth in SGA infants exhibiting lower BWZ (24).

Asymmetric SGA also has different definitions. van der Vlugt et al. regarded the Ponderal index as the definition, that is, (birth weight (g) × 100)/(birth length (cm))^3^ below the 10th percentile (25). Nevertheless, the W/L ratios according to gestational age and sex proposed by the INTERGROWTH-21st Project can better reflect fat mass and fat-free mass (12). Thus, we defined a W/L ratio below the 10th percentile as asymmetric. Multivariate regression analysis showed that asymmetry was not associated with FCUG, which was similar to the length CUG result according to Maciejewski et al. (26).

### Strengths and limitations

#### Strengths

1. A prediction nomogram for SGA catch-up growth failure was established based on a 24-year retrospective cohort, with temporal validation performed using a prospective cohort followed for over 3 years. This model exhibited strong temporal stability.
2. Using perinatal and familial predictors, we developed a prediction nomogram that identifies FCUG risk in SGA children immediately after birth, providing evidence for early clinical intervention.

#### Limitations

1. The SGA infants in this study were not followed up to adulthood. We are working on improving the follow-up system to track the long-term prognosis of SGA infants.
2. West China Second University Hospital is a pediatric research center in southwest China that has a broad range of cases and more comprehensive medical records. Moreover, SGA infants often require referral to higher authorities, and our partner hospitals do not always have extensive data. Therefore, the prediction nomogram has not been externally verified. We are negotiating and expect to cooperate with more hospitals for external verification.
3. This study only focused on whether full-term SGA infants completing catch-up growth. Excessive catch-up growth and long-term cardio-metabolic risks had not been adequately addressed.

## Conclusion

This study developed and validated a prediction nomogram for faltering catch-up growth specifically in full-term SGA infants, using a velocity-based definition that sensitively captures early growth dynamics. The model, built on four easily obtainable perinatal and familial predictors, demonstrated robust performance and offered a practical tool for early risk stratification in clinical settings. Beyond individual prediction, our findings highlight he substantial variability in FCUG incidence arising from heterogeneous definitions of SGA and catch-up growth, underscoring the urgent need for standardized criteria. By combining a clinically feasible prediction tool with a more stringent definition of growth failure, this work provides a novel approach to guiding early monitoring and intervention strategies aimed at improving long-term outcomes in this vulnerable population.

## Data Availability

The raw data supporting the conclusions of this article will be made available by the authors, without undue reservation.
