# Supplementary material for: A prediction nomogram for faltering catch-up growth in full-term small-for-gestational-age infants: a retrospective cohort study
Source: Front Pediatr. 2026 May 18;14:1792285. doi: 10.3389/fped.2026.1792285 (PMC13223142; doi:10.3389/fped.2026.1792285)
Supplement: Supplementary file 1 [file Supplementaryfile1.pdf]

Table S1. The loss of follow-up in SGA infants

Time	Development cohort			Temporal validation cohort		
	Male (n=93)	Female (n=89)	N (n=182)	Male (n=41)	Female (n=45)	N (n=86)
Before three months of age (%)	10(10.8)	10(11.2)	20(11.0)	6(14.6)	18(40.0)	24(27.9)
Three to six months of age (%)	8(8.6)	8(9.0)	16(8.8)	12(29.3)	5(11.1)	17(19.8)
Six to twelve months of age (%)	12(12.9)	16(18.0)	28(15.4)	11(26.8)	11(24.4)	22(25.6)
After twelve months of age (%)	63(67.7)	55(61.8)	118(64.8)	12(29.3)	11(24.4)	23(26.7)

Abbreviations: SGA, small-for-gestational-age

Table S2. The catch-up growth of SGA infants

Age		Development cohort			Temporal validation cohort		
		Male (n=580)	Female (n=605)	N (n=1185)	Male (n=147)	Female (n=147)	N (n=294)
Before three months of age (%)	No	395 (68.2)	396 (65.5)	792 (66.8)	76 (51.7)	81 (55.1)	157 (53.4)
	Yes	184 (31.8)	209 (34.5)	393 (33.2)	71 (48.3)	66 (44.9)	137 (46.6)
Before six months of age (%)	No	238 (41.0)	240 (39.7)	478 (40.3)	46 (31.3)	50 (34.0)	96 (32.7)
	Yes	342 (59.0)	365 (60.3)	707 (59.7)	101 (68.7)	97 (66.0)	198 (67.3)
Before twelve months of age (%)	No	170 (29.3)	173 (28.6)	343 (28.9)	36 (24.5)	41 (27.9)	77 (26.2)
	Yes	410 (70.7)	432 (71.4)	842 (71.1)	111 (75.5)	106 (72.1)	217 (73.8)
Before two years of age (%)	No	146 (25.2)	134 (22.1)	280 (23.6)	28 (19.0)	32 (21.8)	60 (20.4)
	Yes	434 (74.8)	471 (77.9)	905 (76.4)	119 (81.0)	115 (78.2)	234 (79.6)

Abbreviations: SGA, small-for-gestational-age

Table S3. Comparison of models for the prediction of FCUG in SGA infants

Models	Predictors	AIC	AUC	Likelihood-ratio test	
				LogLik	P
Model1	Male sex, BLZ, BWZ, THZ	1042.4	0.808	-516.2	-
Model2	Male sex, BLZ, BWZ, THZ, BLZ:BWZ	1027.9	0.81	-507.96	0.00004917***

"***"Significant difference with P value < 0.05 between model1 and model2 in the likelihood-ratio test.

Abbreviations: FCUG, faltering catch-up growth; SGA, small-for-gestational-age; AIC, Akaike information criterion; AUC, Area under the ROC curve
